# A Minimally Invasive Technique for the Retrieval of Fractured Root Tips

**DOI:** 10.7759/cureus.41458

**Published:** 2023-07-06

**Authors:** Muslat A Bin Rubaia’an, Muath Khaled Alotaibi, Aymen A Neyaz

**Affiliations:** 1 College of Dentistry, Riyadh Elm University, Riyadh, SAU; 2 Oral & Maxillofacial Surgery, Presidency of State Security, Riyadh, SAU

**Keywords:** root tip, fractured, third molar, extraction, endodontic files

## Abstract

Exodontia, the removal of a compromised tooth, ideally consists of the painless removal of the tooth or tooth root, with minimal trauma to the surrounding tissues, resulting in complete healing without creating postoperative prosthetic problems. Fractures of the tooth’s root tip during exodontia can be common in some cases, such as in teeth with irregular root morphology or severely decayed teeth. The current article presents a technical report in which endodontic files made it possible to remove a fractured root tip from a maxillary third molar without using force.

## Introduction

Exodontia is a dental treatment consisting of the removal of a compromised tooth from its dental alveolus in the alveolar bone. Exodontia is primarily performed by an oral surgeon; nevertheless, general practitioners and periodontists can also perform tooth extraction procedures as part of their daily practice. Although it is a relatively simple procedure, it is a highly sensitive technique that depends on the operator's skills. The ideal tooth extraction consists of the painless removal of the tooth or tooth root, with minimal trauma to the surrounding tissues, resulting in complete healing without the creation of postoperative prosthetic problems [1,2]. Fractures of the tooth’s root tip during exodontia can be common in some cases, such as in teeth with irregular root morphology or with severely decayed teeth [2]. The use of a poor extraction technique while retrieving the fractured root with conventional instruments is invasive and can lead to fracture of the buccal cortical plate, fragmentation of bone around the socket, or it can be pushed into adjacent spaces such as the maxillary sinus or inferior alveolar nerve canal [3,4]. The present article describes a case in which endodontic files facilitated the atraumatic removal of a fractured root tip of the maxillary third molar.

## Technical report

**Figure 1:**
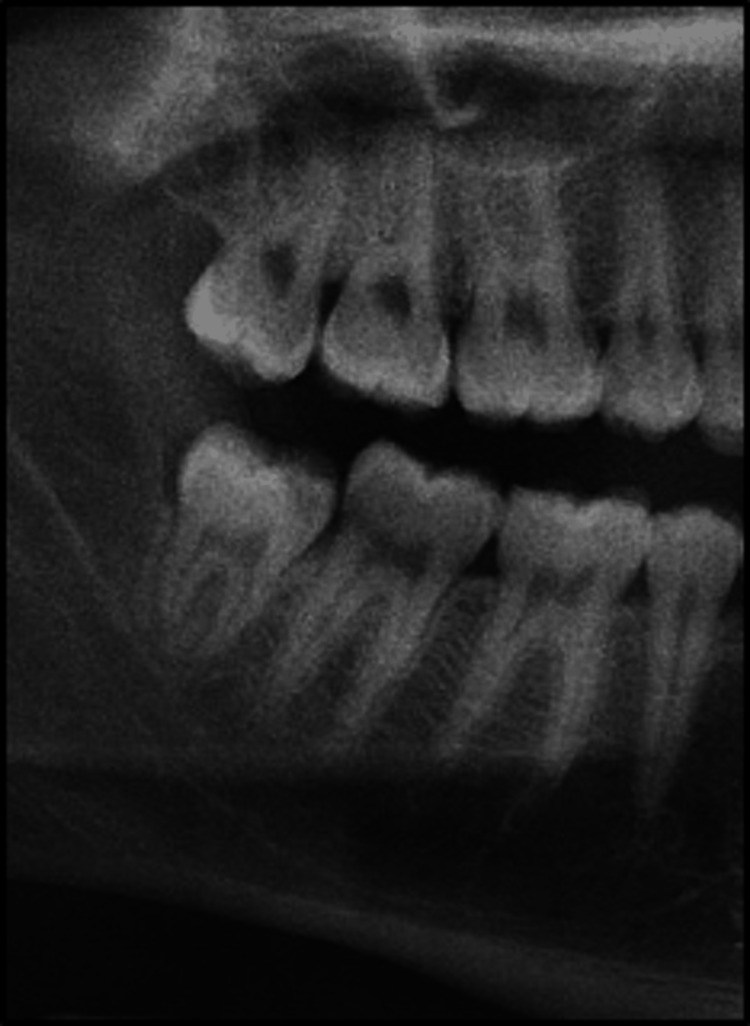
Preoperative cropped orthopantomogram

A 24-year-old male patient presented with the chief complaint of pain in the mandibular right posterior region of the jaw for the past two to three months. Full medical history was recorded, in which the patient was medically fit. He was not on any medication and was unaware of any allergies. Clinical extraoral examination of the temporomandibular joint (TMJ), regional lymph nodes, face symmetry, and muscles of mastication was done without detecting any abnormalities. Clinical intraoral and radiographic examination revealed a carious, partially erupted right mandibular third molar with clinical signs and recurrent symptoms of chronic pericoronitis and non-functioning upper right maxillary third molar after extraction of the opposing tooth and extraction of both mandibular and maxillary third molars was advised as shown in Figure 1.

**Figure 2:**
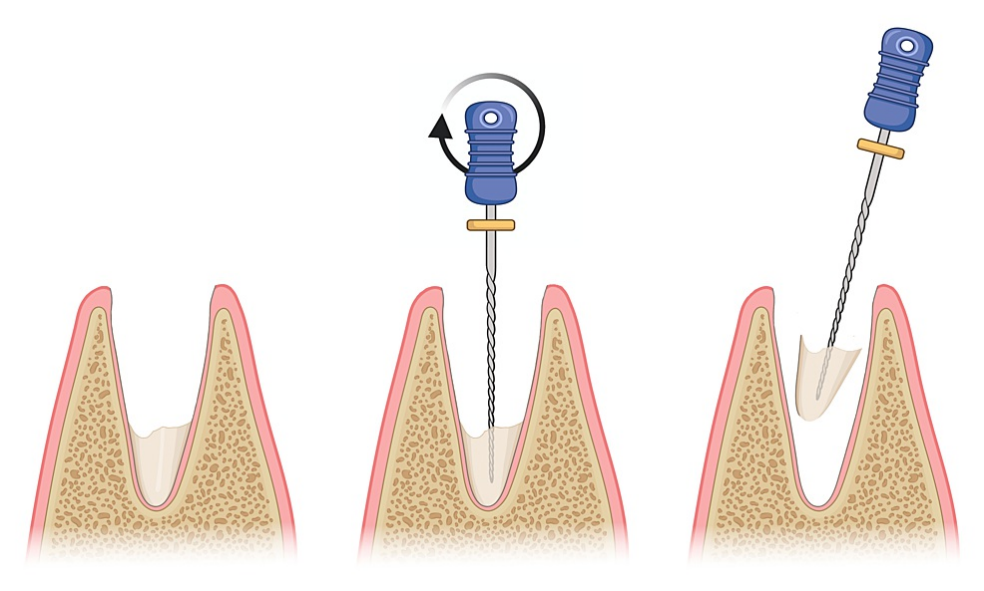
Retrieval of a fractured root tip using an endodontic file Image created with BioRender.com

The palatal root tip was fractured during the maxillary third molar removal. An atraumatic removal was performed by inserting an endodontic file into the canal, screwing it, and pulling it out after it engaged tightly (Figure 2).

**Figure 3:**
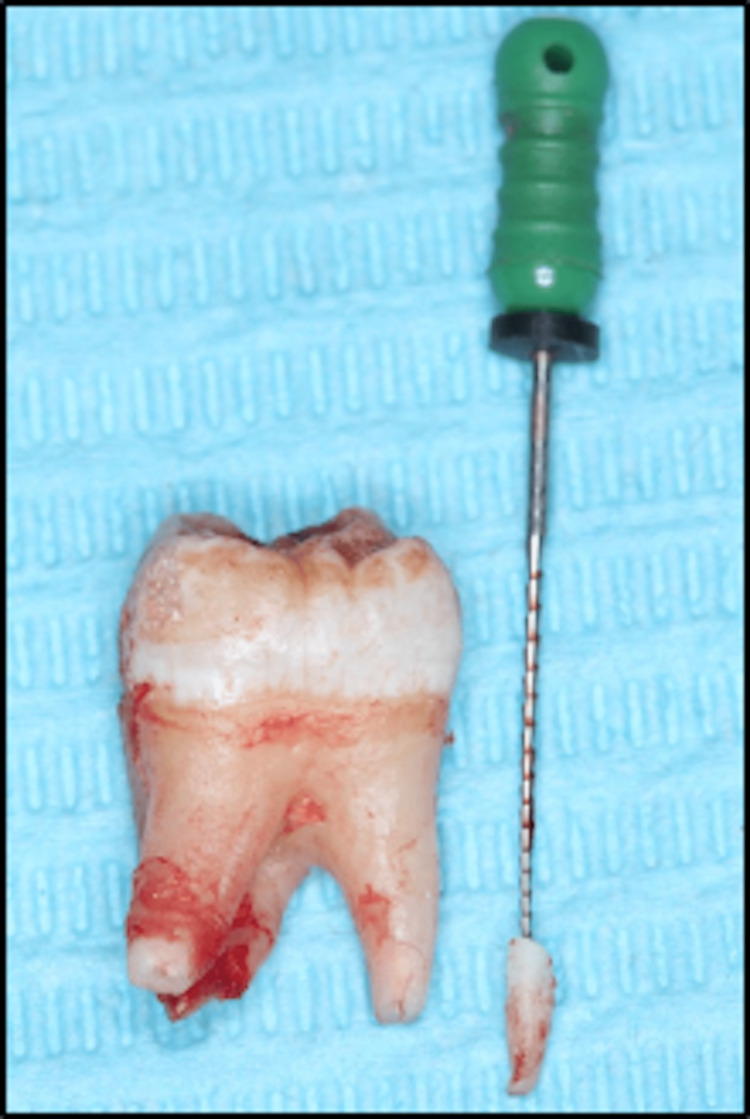
Extracted fragment of the root tip

The fractured root tip was successfully secured and removed with the help of the endodontic file (Figure 3).

**Figure 4:**
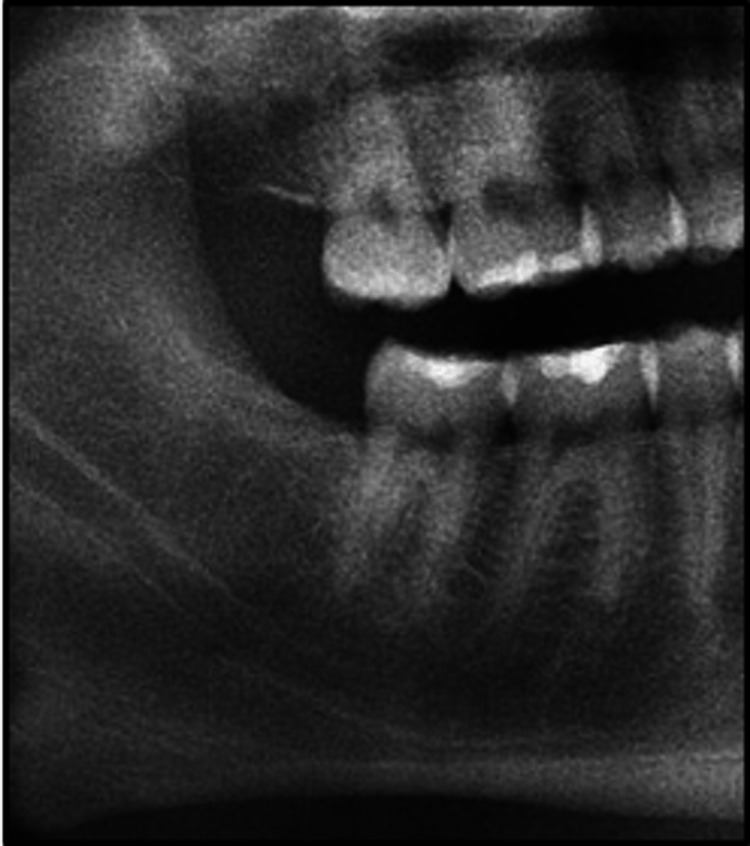
Postoperative cropped orthopantomogram at eight-month follow-up showing adequate healing

An eight-month clinical and radiographical follow-up revealed a normal alveolar bone level (Figure 4).

## Discussion

Exodontia aims to preserve the buccal and lingual or palatal cortical plates and thus should be minimally invasive [2]. Hearing a cracking sound during extraction is usual and normal, as it indicates the yielding of alveolar bone to apical pressure. However, after removing the tooth from its socket, it can sometimes be observed that the root tip has fractured and is retained in the socket. This is a delicate and potentially dangerous situation. A number of techniques have been documented in the literature for removing a fractured root tip, including the creation of a bony window; surgical removal of bone around the root within the socket; and removal with apex elevators, Periotomes, Luxators, and syringe needles [1,5,6]. Endodontic files, particularly H-files, have been used for this purpose and can be considered as one of the most convenient techniques for retrieval. Due to the fact that they have an adhesion force when inserted into the root canal, the root fragment can be secured and removed [2]. Endodontic files are used for cleaning and shaping the root canal. Conventional hand endodontic files, Hedstrom files (H-files), and Kerr files (K-files) are commonly made from stainless steel. They are affordable and available at all dental facilities. H-files have a superior, effective engagement in the dentin than K-files or reamers. H-files engage more efficiently with the root than K-files since they have more contact area within the canal [7]. The size of the file should be approximate to the size of the canal, allowing the file to engage firmly. ISO size files ranging from 20 to 30 can be considered the standard for most teeth. In addition, most roots were retrieved using size 25 files [2]. In case of a few failed attempts to deliver the fragment, the file can be luted with resin-modified glass ionomer cement to gain benefit from the bonding strength [7]. The use of endodontic files for exodontia has numerous advantages over conventional techniques, such as preservation of the surrounding bone, absence of gross anatomical disturbance, decreased operating time, no requirement for additional assisting personnel, patient satisfaction, elimination of the apprehension associated with the noise and vibration of surgical devices, and the fact that it is a relatively straightforward technique to carry out [6]. However, care must be taken when using this technique. The root canal must be visualizable to ensure the file is inserted into the canal rather than the periodontal ligament or the socket itself. The dental floss should be tight to the file to avoid aspiration. Excessive apical pressure should be avoided to prevent displacing the fragment into the maxillary sinus.

## Conclusions

Retrieving fractured root tips using endodontic files is a simple, easy, and effective technique associated with the least amount of trauma to and discrepancy with the surrounding tissue and structures, upholding the goal of minimally invasive dentistry.
